# Principles and Innovations in Peritoneal Surface Malignancy Treatment

**DOI:** 10.4021/wjon660w

**Published:** 2013-07-15

**Authors:** Kelly M. MacArthur, Michael B. Nicholl

**Affiliations:** a Department of Surgery, University of Missouri School of Medicine, Columbia, Missouri 65212, USA; b Ellis Fischel Cancer Center, University of Missouri School of Medicine, Columbia, Missouri 65212, USA

**Keywords:** Pseudomyxoma peritonei, Carcinomatosis, Peritoneal surface malignancy, Cytoreductive surgery, HIPEC

## Abstract

Cytoreductive surgery with heated intraperitoneal chemotherapy (CRS/HIPEC) remains a controversial treatment for malignant disease of the peritoneal cavity. We review the scientific principles underscoring the rationale for CRS/HIPEC, recent innovations and ongoing controversies. Lack of level 1 data limits the understanding of the true benefit of CRS/HIPEC.

## Introduction

Peritoneal carcinomatosis (PC) is a malignant condition that is a result of tumor metastasis within the peritoneal cavity. Peritoneal tumor implants are considered locoregional metastasis, a concept that differentiates PC from other patterns of metastasis and underlies the rationale for aggressive treatment of the disease. Clinically, PC may manifest as malignant ascites, intractable pain, or bowel ischemia/hemorrhage/obstruction/perforation. Commonly, the primary source for PC is a gastrointestinal (GI) or gynecologic (GYN) malignancy [1-3]. The malignant process may also arise from the peritoneum itself, as with primary peritoneal carcinoma (PPC) or malignant peritoneal mesothelioma (MPM) [4] (Table 1). PC is associated with high morbidity, mortality, and poor quality of life. EVOCAPE I reported median survival rates for locoregionally advanced gastric cancer and colorectal cancer were 3.1 months and 5.2 months [5], respectively, while the median survival of patients with stage IV ovarian cancer is 12 - 23 months [6-8]. Poor survival is the rule with primary peritoneal tumors, as well [9].

**Table 1:** Characterization of Malignancy Causing PC Amenable to CRS/HIPEC Treatment [79]

Anatomic origin
Intra-abdominal: gastric, colorectal, pancreatic, hepatocellular, appendiceal
Gynecologic: ovarian, endometrial, uterine
Peritoneum
Renal, bladder
Primary Tumor Histology-Those malignancies capable of spreading to involve the peritoneum are diverse and include
Primary peritoneal carcinomas
Malignant peritoneal mesothelioma
Metastases from a non-peritoneal primary: adenocarcinomas, sarcomas, neuroendocrine tumors, desmoplastic tumors, lymphomas
Pseudomyxoma Peritonei

In the United States, about 250,000 patients yearly are diagnosed with tumors which have the potential to develop PC; perhaps curiously, the majority of these patients will not develop peritoneal disease, which raises questions regarding the molecular mechanisms governing PC development, and, as corollary, raises questions regarding how to predict or provide prophylaxis against PC development. For many years, the standard treatment for PC of GI origin was palliative systemic chemotherapy, with surgery employed only to treat malignancy related complications [10]. Recently, cytoreductive surgery followed by hyperthermic intraperitoneal chemotherapy (CRS/HIPEC) has been advocated to improve outcomes in PC by enhancing both patient survival and quality of life [11]. Vital to the success of this aggressive approach is an understanding of the natural history of regionally confined metastasis [12].

## Rationale of Intraperitoneal Chemotherapy

Systemic chemotherapy administration for PC is minimally effective [12], even when combined with CRS. Adequate drug concentrations cannot be safely achieved in the peritoneal cavity when chemotherapy is administered intravenously (IV) and PC is largely resistant to the low intraperitoneal (IP) concentrations achieved with systemic chemotherapy [12]. Drug penetration from plasma to tumor deposits within the peritoneal cavity is poor at best and even worse when malignant ascites is present [13]. An understanding of the mechanism of failure of systemic chemotherapy for PC has helped foster the development of CRS/HIPEC.

Intraperitoneal chemotherapy (IPEC) permits regional administration of chemotherapy at a concentrated dose, providing greater cytotoxic effect on tumor cells and decreased systemic cytotoxicity via localized delivery [12]. Where the plasma-peritoneal barrier inhibits attainment of effective IP concentration with systemic chemotherapy administration, IPEC utilizes this obstacle in its favor as the physical barrier allows maintenance of localized therapeutic drug concentration levels [14, 15]. Low peritoneal drug clearance is attributable primarily to the nature of the capillary wall which serves to resist large molecule transfer, and secondarily to the peritoneal mesothelium and interstitium which function in a similar manner. With IPEC, the IP to plasma area under concentration (AUC) time curve gradient ratio has the potential to exceed a factor of 1,000, indicative of the pharmacokinetic advantage of this route of administration.

Additionally, the lack of first pass effect contributes to the superior cytotoxicity achieved with IPEC. After IPEC administration, the portal vein transports absorbed chemotherapeutic agent to the liver where the drug undergoes hepatic extraction, thus decreasing systemic drug exposure. Because the chemotherapeutic agent follows the same drainage pathway as tumor cells, hepatic micrometastases in theory may be exposed to cytotoxic drug levels, presenting an additional means of therapy [16]. Some drugs are preferentially transported via the lymphatic system before reaching systemic circulation, thus attaining elevated lymph to plasma drug AUC ratio. Drugs that achieve high lymph AUC ratios may be superior for primary malignancies that disseminate preferentially along lymphatics.

## Rationale of Hyperthermia and Cytoreductive Surgery

Hyperthermia alone is a poor treatment modality for malignancy, but as De Bree et al have remarked, it is a valuable adjunct to IPEC [17]. The major benefit of hyperthermia is the synergistic increase in cytotoxicity with administration of heated chemotherapeutics. The degree of synergism is drug dependent with the highest thermal enhancement ratios achieved with alkylating agents (Melphalan/Cyclophosphamide/Ifosfamide) [18].

In vitro and in vivo studies have demonstrated improved therapeutic index and cytotoxic efficacy of chemotherapeutic drugs when delivered in the setting of HIPEC. Hyperthermia enhances cellular uptake of neoplastic drugs by increasing membrane permeability and membrane transport [19]. Elevated temperatures selectively target malignant cells thereby enhancing the efficacy and specificity of IP drug delivery and increasing the chemotherapeutic activity and tissue penetration [19-23]. The reversible, nonselective cytotoxic mechanism of hyperthermia has been attributed to RNA synthesis inhibition and mitosis arrest. Selective cytotoxic effects are due to acceleration of cell death via alteration of metabolism, increase of lysosome number and function, and modification of microcirculation within malignant cells [19, 24, 25]. Hyperthermia induces inhibition of oxidative metabolism within malignant cells causing accumulation of lactic acid as a result of anaerobic glycolysis. Lysosomal function enhanced by increased intracellular acidity further augments malignant tissue destruction [25]. Dissimilar microcirculatory response of normal and malignant tissue aids the selectivity conferred by hyperthermic treatment: the former demonstrates increased blood flow capacity, while decreased blood flow capacity, to the extent of complete vascular stasis, occurs in the latter [26]. Just as the regional administration of chemotherapy in IPEC limits systemic drug toxicity and the associated side effects, regional thermal application prevents the adverse effects associated with whole body hyperthermia.

The efficacy of HIPEC is enhanced by successful CRS. The combined treatment modality of CRS/HIPEC is superior to IPEC or CRS alone. A recent multi-center analysis evaluating prognosis of patients undergoing CRS/HIPEC for PC of GI origin showed significant survival benefits for patients undergoing this therapy and concluded this dual treatment approach to be the gold standard treatment for PC [27]. Tissue penetration of heat declines within the first few millimeters of application, thereby necessitating adequate CRS [28]. CRS alone is insufficient, as patients are likely to have microscopic residual disease leading to recurrence. Successful completion of macroscopic CRS is a strong predictor of survival and recurrence in CRS/HIPEC [4, 29].

Chua et al investigated the cost-effectiveness of CRS with HIPEC and declared “this complex surgical treatment results in significant increases in medical costs with a parallel increase in survival for a disease that has been poorly treated, and hence may be considered as cost-effective given the observed life years gained” [30]. Despite the promising outcomes of CRS/HIPEC, this regimen has yet to be universally acknowledged as standard treatment for PC. Many insurance providers, including Medicare, still consider the treatment to be experimental when used to treat PC of non-PMP origin and therefore may cover very little of the cost [31].

## Morbidity, Mortality, Toxicity of CRS/HIPEC

The morbidity, mortality and toxicity of CRS/HIPEC are major challenges to treating PC; however, perioperative morbidity of 33-43% and mortality of 3-4% are within expectations for major GI surgery [32]. The extent of peritoneal involvement, extent of organ resection, institutional experience, and patient age all factor into the occurrence of complications. Major complications specific to CRS/HIPEC include GI fistulas [33], fluid-electrolyte shifts with resulting metabolic and acid-base imbalance [34], pulmonary complications, and chemotherapy-related hematologic and renal toxicities. Even patients with uncomplicated post-operative recovery will display minor physiologic derangements [35].

As would be expected, severe post-operative complications increase the complexity of post-operative care and increase length of hospitalization [36]. Chua et al noted the great importance of appropriate patient selection thereby limiting treatment to those patients who are most likely to achieve long-term survival and sparing those with little therapeutic likelihood from major complications [37]. Despite the risks associated with CRS/HIPEC, it is essential to consider the potential benefits and improved long-term outcomes obtained with CRS/HIPEC as compared to less aggressive treatment for PC.

## Clinical Application of CRS/HIPEC

The commonly accepted indication for CRS/HIPEC is treatment of pseudomyxoma peritonei (PMP) [38]. PMP is a rare clinical entity resulting from dissemination of a low-grade mucinous appendiceal or ovarian cystic neoplasm, colloquially known as “jelly belly” [39]. While the clinical course is indolent, untreated PMP is fatal. Investigation of disease specific factors affecting treatment outcomes led to recognition that PMP’s perioperative morbidity and mortality is sufficiently low to be deemed acceptable when operative procedures are adequately short; bulky disease necessitates longer operation yielding greater risk of complications that may outweigh treatment benefits [40]. Overall, the introduction of CRS/HIPEC has changed the natural history of PMP [41].

Beyond PMP, the indications for CRS/HIPEC remain controversial and passionately debated. PC in colorectal cancer CRC is common and its presence yields an unfavorable prognosis, even amongst CRC patients with metastatic disease stratified by site of metastasis [42]. PC accounts for 13% CRC deaths, making progression within the peritoneum the second most common cause of CRC mortality [43-45]. Improved survival with aggressive combined therapy for visceral metastasis of CRC is the prototype for treatment of locoregional metastasis and a model for CRS/HIPEC for PC in CRC [46]. Encouraging evidence for CRS/HIPEC in CRC PC comes from published median and five-year survival rates (48 - 63 months and 51%) which compare favorably to those achieved with systemic chemotherapy (5 - 13 months and 13%) [47-50]. However, the enthusiasm for CRC/HIPEC must be tempered by the lack of good scientific support. To date the only prospective randomized data for CRS/HIPEC in CRC comes from Verwaal et al. In this study, which randomized patients to CRS/HIPEC or systemic 5-FU/LV, a significant survival advantage was demonstrated for HIPEC in the initial publication and the follow-up at 8 years (12.6 month vs 22.2 month median disease free survival) [51, 52]. The major criticism of this study resides in the applicability of the data in the current era of effective chemotherapy [53]. The relatively high surgical mortality (8%) is also a point of contention. While the enthusiasm for CRS/HIPEC for colorectal PC continues to grow as evidenced by the explosion of relevant literature, the true effectiveness of this treatment regimen remains unknown due to limitations in the data (often low powered and nonrandomized) [54].

A common denominator in HIPEC studies is the effect of complete cytoreduction on outcomes. Greater than 25% long term survival with 33% mortality within five years (secondary to malignancy recurrence) was reported following complete CRS with HIPEC for peritoneal colorectal carcinomatosis, whereas significantly worse long term outcomes were associated with incomplete macroscopic resection [29]. Refined patient selection might increase the likelihood of complete cytoreduction and ultimately the success in CRC. Investigators have explored the role of CRS/HIPEC in patients lacking definitive preoperative diagnosis of PC, but at high risk of intra-abdominal CRC recurrence. Due to limitations of early PC detection via conventional imaging, tumor markers, or symptoms, PC is often not discovered until later stages, when complete cytoreduction is less feasible. Allowing early detection and intervention of PC, mandatory second look surgery within those at greater risk for peritoneal spread (Table 2) improves long-term survival due to greater likelihood of complete resection of diseased peritoneum. A randomized trial is currently gathering data to assess treatment of patients with high risk of colorectal carcinomatosis development (Table 2). Pending results of this prospective trial comparing the outcomes of mandatory second look laparotomy with CRS/HIPEC versus standard of care (routine surveillance), utility of surgical intervention could be validated for patients with preoperatively undetectable peritoneal disease but at high risk for metastatic involvement [55].

**Table 2:** Criteria for Patients Found to be at Greater Risk of Colorectal PC Development; Prospective Study’s Defined Criteria for Patients With High Risk of Colorectal PC Development [55]

Criteria for patients found to be at greater risk of colorectal PC development [80]
Symptoms/signs of disease recurrence
Increased risk for regional malignancy recurrence
Rising CEA blood level
Prospective study’s defined criteria for patients with high risk of colorectal PC development [55]
Ovarian metastases
Emergent signs of obstruction or bleeding
Tumor perforation
Limited/resected disease on initial surgery
T4 lesions with adjacent organ resection en bloc

55% of such high risk patients meeting one of the below criteria will develop PC.

There is no level 1 support for the use of CRS/HIPEC therapy in PC of gastric or ovarian origin; however ongoing clinical trials have shown great promise in demonstrating the utility of CRS/HIPEC for select PC patients with aforementioned primary tumors [56, 57]. Recently a survival advantage has been shown for gastric carcinomatosis treated with CRS/HIPEC. CRS/HIPEC has also shown utility for control of ascites secondary to gastric carcinoma [58]. In a multi-institutional study, improved long-term survival was afforded by treatment with CRS and perioperative intraperitoneal chemotherapy when strict patient selection was employed, with noted high mortality rate when selection criteria were not met. Patient and environmental factors (selection of those with limited, resectable gastric carcinomatosis and only “experienced institution” involvement of patient care, respectively) were found to contribute to such criteria [59].

Described as the cornerstone of ovarian cancer treatment [60], cytoreduction with or without chemotherapy administration has proven benefit in the management of advanced primary and recurrent ovarian cancer. Complete cytoreduction is key to increasing survival and improving quality of life [61]. Ovarian cancer has a high likelihood of local recurrence since most patients present at an advanced stage of disease [61]. Even in the setting of ovarian cancer recurrence, repeat CRS can be performed, although with diminishing benefits [62]. With the success of CRS, it is a logical next step to consider incorporation of CRS/HIPEC into ovarian cancer treatment, although the approach has had a difficult time gaining acceptance [63]. In patients with aggressively pretreated, recurrent ovarian cancer, CRS/HIPEC can significantly impact overall survival, with acceptable morbidity rates. As with PC of GI origin, appropriate patient selection is imperative: biologically relatively favorable malignancy and feasible macroscopically complete resection are main factors to optimize outcomes [64]. Thus, epithelial ovarian cancer has become increasingly recognized as a chronic condition with high risk of recurrences but potentially amenable to repeat CRS/HIPEC therapies. Phase II studies have validated the CRS/HIPEC treatment of PC secondary to advanced primary, recurrent, and platinum resistant disease [65, 66]; multiple randomized phase III trials are currently accruing patients in order to understand if CRS/HIPEC has a place in ovarian cancer treatment [60, 63, 67].

## Laparoscopic CRS/HIPEC

A minimally invasive approach to CRS/HIPEC is appealing if many of the advantages of laparoscopic surgery (less pain, shorter hospital stay, less blood loss, better cosmesis, etc) translate to management of peritoneal surface malignancy. Data are accumulating in regards to this approach [68], but are still very limited. In distinction to therapeutic intervention, laparoscopy already plays an important, albeit occasional, role in diagnosis and in staging [69, 70], since most information for surgical planning can be obtained from axial imaging and percutaneous biopsy [71, 72]. The technical challenges of minimally invasive cytoreduction have been investigated and, in particular, laparoscopic peritonectomy is technically feasible [73]. Preclinical models also suggest increased intraabdominal pressure associated with laparoscopy effects the pharmacokinetics of HIPEC agents resulting in higher systemic, tumor and normal tissue absorption of drug [74]. The clinical implication of this effect is unknown. Clinical applications are probably best suited for low-volume disease [75] and in the setting of second look operations for high-risk disease [76].

## Conclusion

Management of peritoneal metastases has proved challenging, largely due to the complexity and incomplete understanding of the pathobiology of PC. HIPEC in combination with CRS, has gained in popularity since its early innovation [77], however, concerns regarding the complications of CRS/HIPEC, inaccuracies of imaging to properly detect extent of invasion translating to an inaccuracy of patient selection, and lack of familiarity and surgeon training with HIPEC, have kept this therapy regimen from becoming standard of treatment. The narrow understanding of molecular interactions underlying the development of peritoneal surface malignancy has limited the discovery of molecular prognostic markers and hindered the development of preventive and molecular targeted therapeutics [78]. The controversies surrounding the efficacy and utility of such treatment, resulting in a general lack of acceptance of such a therapeutic combination for a select group of advanced disease cancer patients, have not quelled the development of further medical developments. However, in the event that additional studies validate the benefit of such treatments in enhancing HIPEC treatment of peritoneal carcinomatosis, the hesitation to accept utility of CRS with HIPEC could potentially threaten the acceptance of these and other novel, promising medical treatment options on the horizon.

## References

[1] Suleiman A UN, Al-Imam O, Khuliefat S, Al-Sakran M (2004). Peritoneal Carcinomatosis computerized tomography scans findings and causes. JRMS.

[2] Levitt RG, Koehler RE, Sagel SS, Lee JK (1982). Metastatic disease of the mesentery and omentum. Radiol Clin North Am.

[3] Nelson RC, Chezmar JL, Hoel MJ, Buck DR, Sugarbaker PH (1992). Peritoneal carcinomatosis: preoperative CT with intraperitoneal contrast material. Radiology.

[4] Glockzin G, Schlitt HJ, Piso P (2009). Peritoneal carcinomatosis: patients selection, perioperative complications and quality of life related to cytoreductive surgery and hyperthermic intraperitoneal chemotherapy. World J Surg Oncol.

[5] Sadeghi B, Arvieux C, Glehen O, Beaujard AC, Rivoire M, Baulieux J, Fontaumard E (2000). Peritoneal carcinomatosis from non-gynecologic malignancies: results of the EVOCAPE 1 multicentric prospective study. Cancer.

[6] Hardy JR, Wiltshaw E, Blake PR, Harper P, Slevin M, Perren TJ, Tan S (1991). Cisplatin and carboplatin in combination for the treatment of stage IV ovarian carcinoma. Ann Oncol.

[7] Curtin JP, Malik R, Venkatraman ES, Barakat RR, Hoskins WJ (1997). Stage IV ovarian cancer: impact of surgical debulking. Gynecol Oncol.

[8] Akahira JI, Yoshikawa H, Shimizu Y, Tsunematsu R, Hirakawa T, Kuramoto H, Shiromizu K (2001). Prognostic factors of stage IV epithelial ovarian cancer: a multicenter retrospective study. Gynecol Oncol.

[9] Sebbag G, Shmookler BM, Chang D, Sugarbaker PH (2001). Peritoneal carcinomatosis from an unknown primary site. Management of 15 patients.Tumori.

[10] Glockzin G, Ghali N, Lang SA, Agha A, Schlitt HJ, Piso P (2007). [Peritoneal carcinomatosis. Surgical treatment, including hyperthermal intraperitoneal chemotherapy]. Chirurg.

[11] Esquivel J, Sticca R, Sugarbaker P, Levine E, Yan TD, Alexander R, Baratti D (2007). Cytoreductive surgery and hyperthermic intraperitoneal chemotherapy in the management of peritoneal surface malignancies of colonic origin: a consensus statement. Society of Surgical Oncology. Ann Surg Oncol.

[12] de Bree E, Tsiftsis DD (2007). Principles of perioperative intraperitoneal chemotherapy for peritoneal carcinomatosis. Recent Results Cancer Res.

[13] Sugarbaker PH, Stuart OA, Vidal-Jove J, Pessagno AM, DeBruijn EA (1996). Pharmacokinetics of the peritoneal-plasma barrier after systemic mitomycin C administration. Cancer Treat Res.

[14] Jacquet P, Sugarbaker PH (1996). Peritoneal-plasma barrier. Cancer Treat Res.

[15] Flessner MF (2005). The transport barrier in intraperitoneal therapy. Am J Physiol Renal Physiol.

[16] Speyer JL, Sugarbaker PH, Collins JM, Dedrick RL, Klecker RW, Myers CE (1981). Portal levels and hepatic clearance of 5-fluorouracil after intraperitoneal administration in humans. Cancer Res.

[17] de Bree E, Romanos J, Tsiftsis DD (2002). Hyperthermia in anticancer treatment. Eur J Surg Oncol.

[18] Takemoto M, Kuroda M, Urano M, Nishimura Y, Kawasaki S, Kato H, Okumura Y (2003). The effect of various chemotherapeutic agents given with mild hyperthermia on different types of tumours. Int J Hyperthermia.

[19] Sticca RP, Dach BW (2003). Rationale for hyperthermia with intraoperative intraperitoneal chemotherapy agents. Surg Oncol Clin N Am.

[20] Storm FK (1989). Clinical hyperthermia and chemotherapy. Radiol Clin North Am.

[21] Panteix G, Guillaumont M, Cherpin L, Cuichard J, Gilly FN, Carry PY, Sayag A (1993). Study of the pharmacokinetics of mitomycin C in humans during intraperitoneal chemohyperthermia with special mention of the concentration in local tissues. Oncology.

[22] Jacquet P, Averbach A, Stuart OA, Chang D, Sugarbaker PH (1998). Hyperthermic intraperitoneal doxorubicin: pharmacokinetics, metabolism, and tissue distribution in a rat model. Cancer Chemother Pharmacol.

[23] Benoit L, Duvillard C, Rat P, Chauffert B (1999). [The effect of intra-abdominal temperature on the tissue and tumor diffusion of intraperitoneal cisplatin in a model of peritoneal carcinomatosis in rats]. Chirurgie.

[24] Cavaliere R, Ciocatto EC, Giovanella BC, Heidelberger C, Johnson RO, Margottini M, Mondovi B (1967). Selective heat sensitivity of cancer cells. Biochemical and clinical studies.Cancer.

[25] Overgaard J (1977). Effect of hyperthermia on malignant cells in vivo. A review and a hypothesis.Cancer.

[26] Dudar TE, Jain RK (1984). Differential response of normal and tumor microcirculation to hyperthermia. Cancer Res.

[27] Elias D, Glehen O, Pocard M, Quenet F, Goere D, Arvieux C, Rat P (2010). A comparative study of complete cytoreductive surgery plus intraperitoneal chemotherapy to treat peritoneal dissemination from colon, rectum, small bowel, and nonpseudomyxoma appendix. Ann Surg.

[28] van Ruth S, Verwaal VJ, Hart AA, van Slooten GW, Zoetmulder FA (2003). Heat penetration in locally applied hyperthermia in the abdomen during intra-operative hyperthermic intraperitoneal chemotherapy. Anticancer Res.

[29] Shen P WG, Stewart JH, McCoy TP, Levine EA (2010). Prognostic Factors for Actual 5-year Survivors after Cytoreductive Surgery and Hyperthermic Intraperitoneal Chemotherapy for Colorectal Cancer with Peritoneal Surface Disease. Journal of Clinical Oncology..

[30] Chua TC, Martin S, Saxena A, Liauw W, Yan TD, Zhao J, Lok I (2010). Evaluation of the cost-effectiveness of cytoreductive surgery and hyperthermic intraperitoneal chemotherapy (peritonectomy) at the St George Hospital peritoneal surface malignancy program. Ann Surg.

[31] Cross IB Hyperthermic Intraperitoneal Chemotherapy (HIPEC) Medical Policy Bulletin.

[32] Chua TC, Yan TD, Saxena A, Morris DL (2009). Should the treatment of peritoneal carcinomatosis by cytoreductive surgery and hyperthermic intraperitoneal chemotherapy still be regarded as a highly morbid procedure?: a systematic review of morbidity and mortality. Ann Surg.

[33] Saxena A, Chua TC, Yan TD, Morris DL (2010). Postoperative pancreatic fistula after cytoreductive surgery and perioperative intraperitoneal chemotherapy: incidence, risk factors, management, and clinical sequelae. Ann Surg Oncol.

[34] Raft J, Parisot M, Marchal F, Tala S, Desandes E, Lalot JM, Guillemin F (2010). [Impact of the hyperthermic intraperitoneal chemotherapy on the fluid-electrolytes changes and on the acid-base balance]. Ann Fr Anesth Reanim.

[35] Elias D, Di Pietrantonio D, Boulet T, Honore C, Bonnet S, Goere D, Kohneh-Shahri N (2009). "Natural history" of complete cytoreductive surgery with hyperthermic intraperitoneal chemotherapy. Eur J Surg Oncol.

[36] Ahmad SA, Kim J, Sussman JJ, Soldano DA, Pennington LJ, James LE, Lowy AM (2004). Reduced morbidity following cytoreductive surgery and intraperitoneal hyperthermic chemoperfusion. Ann Surg Oncol.

[37] Chua TC, Saxena A, Schellekens JF, Liauw W, Yan TD, Fransi S, Zhao J (2010). Morbidity and mortality outcomes of cytoreductive surgery and perioperative intraperitoneal chemotherapy at a single tertiary institution: towards a new perspective of this treatment. Ann Surg.

[38] Vaira M, Cioppa T, G DEM, Bing C, D'Amico S, D'Alessandro M, Fiorentini G (2009). Management of pseudomyxoma peritonei by cytoreduction+HIPEC (hyperthermic intraperitoneal chemotherapy): results analysis of a twelve-year experience. In Vivo.

[39] Qu ZB, Liu LX (2006). Management of pseudomyxoma peritonei. World J Gastroenterol.

[40] Saxena A, Yan TD, Chua TC, Morris DL (2010). Critical assessment of risk factors for complications after cytoreductive surgery and perioperative intraperitoneal chemotherapy for pseudomyxoma peritonei. Ann Surg Oncol.

[41] Sugarbaker PH (1994). Pseudomyxoma peritonei. A cancer whose biology is characterized by a redistribution phenomenon. Ann Surg.

[42] Franko J, Shi Q, Goldman CD, Pockaj BA, Nelson GD, Goldberg RM, Pitot HC (2012). Treatment of colorectal peritoneal carcinomatosis with systemic chemotherapy: a pooled analysis of north central cancer treatment group phase III trials N9741 and N9841. J Clin Oncol.

[43] Jemal A, Siegel R, Ward E (2008). Cancer statistics, 2008. CA: A cancer journal for clinicians.

[44] Jayne DG, Fook S, Loi C, Seow-Choen F (2002). Peritoneal carcinomatosis from colorectal cancer. Br J Surg.

[45] Macri A, Saladino E, Bartolo V (2010). Peritoneal carcinomatosis of colorectal origin. World journal of gastrointestinal oncology.

[46] Tomlinson JS, Jarnagin WR, DeMatteo RP, Fong Y, Kornprat P, Gonen M, Kemeny N (2007). Actual 10-year survival after resection of colorectal liver metastases defines cure. J Clin Oncol.

[47] Elias D, Lefevre JH, Chevalier J, Brouquet A, Marchal F, Classe JM, Ferron G (2009). Complete cytoreductive surgery plus intraperitoneal chemohyperthermia with oxaliplatin for peritoneal carcinomatosis of colorectal origin. J Clin Oncol.

[48] Verwaal VJ, van Ruth S, Witkamp A, Boot H, van Slooten G, Zoetmulder FA (2005). Long-term survival of peritoneal carcinomatosis of colorectal origin. Ann Surg Oncol.

[49] Yan TD, Black D, Savady R, Sugarbaker PH (2006). Systematic review on the efficacy of cytoreductive surgery combined with perioperative intraperitoneal chemotherapy for peritoneal carcinomatosis from colorectal carcinoma. J Clin Oncol.

[50] Elias D, Goere D, Di Pietrantonio D, Boige V, Malka D, Kohneh-Shahri N, Dromain C (2008). Results of systematic second-look surgery in patients at high risk of developing colorectal peritoneal carcinomatosis. Ann Surg.

[51] Verwaal VJ, van Ruth S, de Bree E, van Sloothen GW, van Tinteren H, Boot H, Zoetmulder FA (2003). Randomized trial of cytoreduction and hyperthermic intraperitoneal chemotherapy versus systemic chemotherapy and palliative surgery in patients with peritoneal carcinomatosis of colorectal cancer. J Clin Oncol.

[52] Verwaal VJ, Bruin S, Boot H, van Slooten G, van Tinteren H (2008). 8-year follow-up of randomized trial: cytoreduction and hyperthermic intraperitoneal chemotherapy versus systemic chemotherapy in patients with peritoneal carcinomatosis of colorectal cancer. Ann Surg Oncol.

[53] Sugarbaker PH, Ryan DP (2012). Cytoreductive surgery plus hyperthermic perioperative chemotherapy to treat peritoneal metastases from colorectal cancer: standard of care or an experimental approach?. Lancet Oncol.

[54] Khrizman P MM (2010). Curative Approach for Stage IV Colorectal Cancer with Multiorgan Involvement: What Makes Sense and What Doesn't?. Current Colorectal Cancer Reports.

[55] Ripley RT, Davis JL, Kemp CD, Steinberg SM, Toomey MA, Avital I (2010). Prospective randomized trial evaluating mandatory second look surgery with HIPEC and CRS vs. standard of care in patients at high risk of developing colorectal peritoneal metastases. Trials.

[56] Bartlett DL (2008). HIPEC: the complexities of clinical trials. Ann Surg Oncol.

[57] Davies JM, O'Neil B (2009). Peritoneal carcinomatosis of gastrointestinal origin: natural history and treatment options. Expert Opin Investig Drugs.

[58] Yang XJ, Li Y, Yonemura Y (2010). Cytoreductive surgery plus hyperthermic intraperitoneal chemotherapy to treat gastric cancer with ascites and/or peritoneal carcinomatosis: Results from a Chinese center. J Surg Oncol.

[59] Glehen O, Gilly FN, Arvieux C, Cotte E, Boutitie F, Mansvelt B, Bereder JM (2010). Peritoneal carcinomatosis from gastric cancer: a multi-institutional study of 159 patients treated by cytoreductive surgery combined with perioperative intraperitoneal chemotherapy. Ann Surg Oncol.

[60] Fagotti A GV, Romano F, Fanfani F, Rossitto C, Vizzielli G, Costantini B, Scambia G (2010). Role of cytoreductive surgery in recurrent ovarian cancer. Therapy.

[61] Fagotti A, Gallotta V, Romano F, Fanfani F, Rossitto C, Naldini A, Vigliotta M (2010). Peritoneal carcinosis of ovarian origin. World J Gastrointest Oncol.

[62] Harter P, Hilpert F, Mahner S, Kommoss S, Heitz F, du Bois A (2009). Role of cytoreductive surgery in recurrent ovarian cancer. Expert Rev Anticancer Ther.

[63] Herzog TJ (2012). The role of heated intraperitoneal chemotherapy (HIPEC) in ovarian cancer: hope or hoax?. Ann Surg Oncol.

[64] Ceelen WP, Van Nieuwenhove Y, Van Belle S, Denys H, Pattyn P (2012). Cytoreduction and hyperthermic intraperitoneal chemoperfusion in women with heavily pretreated recurrent ovarian cancer. Ann Surg Oncol.

[65] Bakrin N, Cotte E, Golfier F, Gilly FN, Freyer G, Helm W, Glehen O (2012). Cytoreductive surgery and hyperthermic intraperitoneal chemotherapy (HIPEC) for persistent and recurrent advanced ovarian carcinoma: a multicenter, prospective study of 246 patients. Ann Surg Oncol.

[66] Classe JM, Muller M, Frenel JS, Berton Rigaud D, Ferron G, Jaffre I, Gladieff L (2010). [Intraperitoneal chemotherapy in the treatment of advanced ovarian cancer]. J Gynecol Obstet Biol Reprod (Paris).

[67] Dovern E, de Hingh IH, Verwaal VJ, van Driel WJ, Nienhuijs SW (2010). Hyperthermic intraperitoneal chemotherapy added to the treatment of ovarian cancer. A review of achieved results and complications. Eur J Gynaecol Oncol.

[68] Sommariva A, Zagonel V, Rossi CR (2012). The role of laparoscopy in peritoneal surface malignancies selected for hyperthermic intraperitoneal chemotherapy (HIPEC). Ann Surg Oncol.

[69] Yan TD, Morris DL, Shigeki K, Dario B, Marcello D (2008). Preoperative investigations in the management of peritoneal surface malignancy with cytoreductive surgery and perioperative intraperitoneal chemotherapy: Expert consensus statement. J Surg Oncol.

[70] Garofalo A, Valle M (2009). Laparoscopy in the management of peritoneal carcinomatosis. Cancer J.

[71] Jacquet P, Jelinek JS, Steves MA, Sugarbaker PH (1993). Evaluation of computed tomography in patients with peritoneal carcinomatosis. Cancer.

[72] Spencer JA, Swift SE, Wilkinson N, Boon AP, Lane G, Perren TJ (2001). Peritoneal carcinomatosis: image-guided peritoneal core biopsy for tumor type and patient care. Radiology.

[73] Ferron G, Gesson-Paute A, Classe JM, Querleu D (2005). Feasibility of laparoscopic peritonectomy followed by intra-peritoneal chemohyperthermia: an experimental study. Gynecol Oncol.

[74] Esquis P, Consolo D, Magnin G, Pointaire P, Moretto P, Ynsa MD, Beltramo JL (2006). High intra-abdominal pressure enhances the penetration and antitumor effect of intraperitoneal cisplatin on experimental peritoneal carcinomatosis. Ann Surg.

[75] Esquivel J, Averbach A, Chua TC (2011). Laparoscopic cytoreductive surgery and hyperthermic intraperitoneal chemotherapy in patients with limited peritoneal surface malignancies: feasibility, morbidity and outcome in an early experience. Ann Surg.

[76] Elias D, Honore C, Dumont F, Ducreux M, Boige V, Malka D, Burtin P (2011). Results of systematic second-look surgery plus HIPEC in asymptomatic patients presenting a high risk of developing colorectal peritoneal carcinomatosis. Ann Surg.

[77] Sugarbaker PH, Cunliffe WJ, Belliveau J, de Bruijn EA, Graves T, Mullins RE, Schlag P (1989). Rationale for integrating early postoperative intraperitoneal chemotherapy into the surgical treatment of gastrointestinal cancer. Semin Oncol.

[78] Kusamura S, Baratti D, Zaffaroni N, Villa R, Laterza B, Balestra MR, Deraco M (2010). Pathophysiology and biology of peritoneal carcinomatosis. World J Gastrointest Oncol.

[79] Sugarbaker PH (2011). Second-look surgery for colorectal cancer: revised selection factors and new treatment options for greater success. Int J Surg Oncol.

